# Quantitative comparison of 68-keV monoenergetic, routine-dose, and low-dose 120-kVp CT for calcium-mimicking inserts in thoracic phantoms: effects of phantom size and reconstruction method

**DOI:** 10.1186/s41747-026-00818-8

**Published:** 2026-09-30

**Authors:** Yeu-Sheng Tyan, Ya-Yun Hsiao, Cheng-Chang Lu, Tou-Rong Chen

**Affiliations:** 1 Department of Medical Imaging, Chung Shan Medical University Hospital, Taichung, 402 Taiwan; 2 Department of Medical Imaging and Radiological Sciences, Chung Shan Medical University, Taichung, 402 Taiwan; 3 School of Medicine, Chung Shan Medical University, Taichung, 402 Taiwan; 4 Center for Fundamental Sciences, National Formosa University, Yunlin, 632 Taiwan; 5 Department of Radiation Oncology, Chung Shan Medical University Hospital, Taichung, 402 Taiwan

**Keywords:** Calcium-mimicking insert, Deep-learning reconstruction, Dual-energy CT, Low-dose CT, Monoenergetic imaging

## Abstract

**Objective:**

68-keV monoenergetic images from dual-energy CT are often compared with conventional 120-kVp CT, but quantitative performance may depend on phantom size, reconstruction method, and dose settings. We performed a technical phantom-based comparison of 68-keV monoenergetic, routine-dose, and low-dose 120-kVp CT for calcium-mimicking inserts.

**Materials and methods:**

In this phantom study, a thorax phantom with a cardiac calcification insert was scanned using routine-dose 120-kVp CT, low-dose 120-kVp CT, and dual-energy CT with 68-keV monoenergetic reconstruction. Small- and large-phantom conditions were created without and with an extension ring. Images were reconstructed at 2.5-mm slice thickness using filtered back projection (FBP), iterative reconstruction (IR), and deep-learning reconstruction (DL). Mean attenuation, background noise, contrast-to-noise ratio (CNR), and figure of merit (FOM, CNR²/CTDIvol) were summarized descriptively. Repeated scans and repeated region of interest redraws were not performed.

**Results:**

For both 5-mm and 3-mm inserts, attenuation generally increased with hydroxyapatite (HA) density. The with-ring condition showed higher background noise and lower CNR. DL showed the most favorable descriptive quantitative results. Under most conditions, 68-keV imaging showed the highest CNR, followed by routine-dose and low-dose 120-kVp CT; however, it was also associated with the highest CTDIvol. When the dose was evaluated using FOM, the differences among the three strategies were smaller.

**Conclusion:**

In this technical phantom study, 68-keV monoenergetic imaging showed a descriptively higher CNR than routine-dose and low-dose 120-kVp CT, but this was accompanied by a higher radiation dose. Dose-normalized performance was more comparable across strategies. Findings should be interpreted as descriptive condition-level comparisons rather than statistically tested superiority.

**Key Points:**

***Question*** How do 68-keV monoenergetic, routine-dose 120-kVp, and low-dose 120-kVp CT differ in conspicuity and dose-normalized performance for calcium-mimicking targets?

***Findings*** 68-keV imaging generally yielded the highest CNR, but also the highest CTDIvol; when dose was considered using FOM, performance differences became smaller.

***Relevance statement*** This phantom study provides a technical framework for calcium-focused CT protocol optimization by comparing conspicuity, radiation dose, and dose-normalized performance across monoenergetic and conventional acquisitions.

**Graphical Abstract:**

**Figure:**
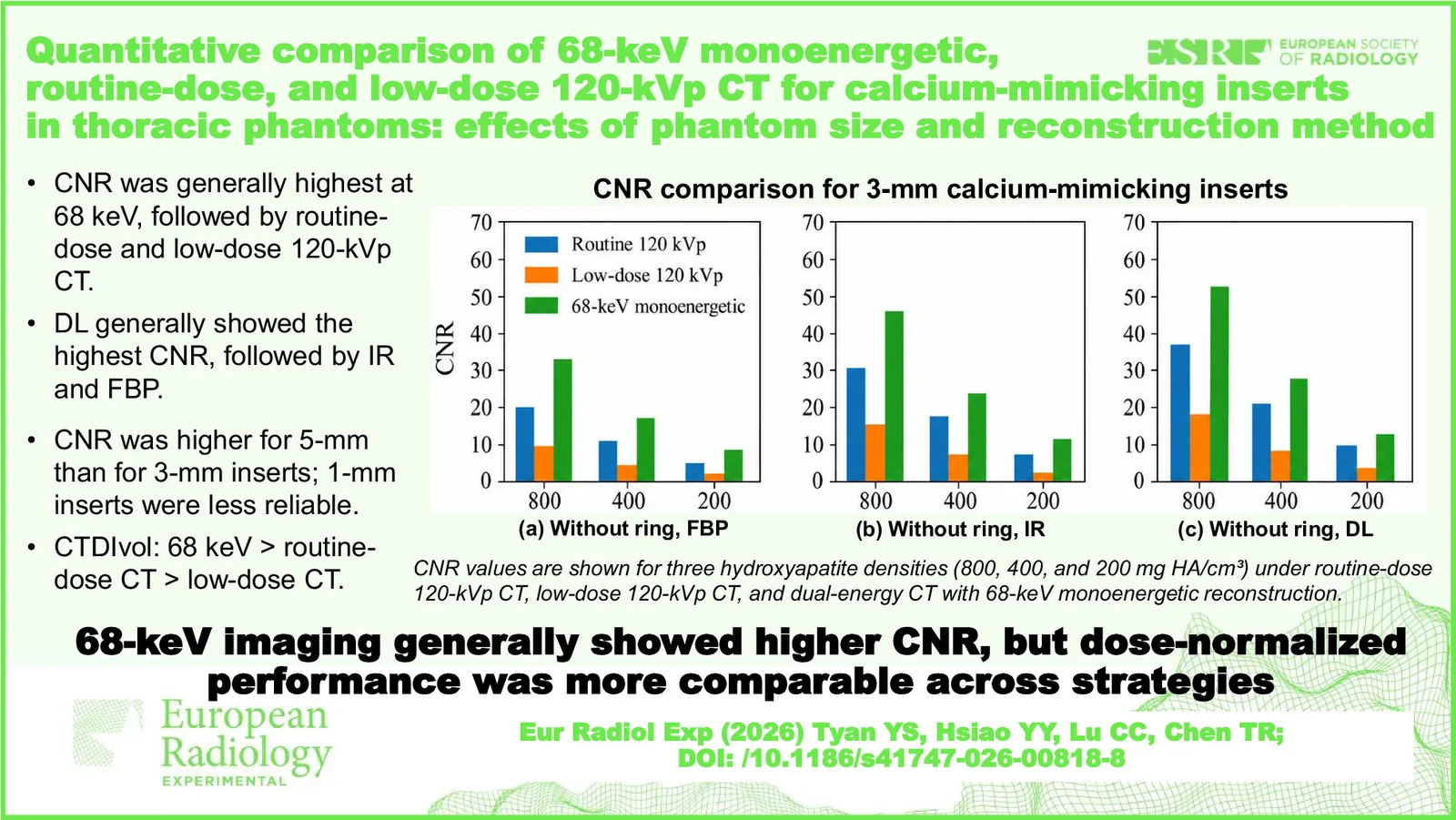

## Background

Coronary and vascular calcifications are important imaging targets because their visibility can influence detection, characterization, and quantitative assessment. In CT, the conspicuity of calcified structures depends not only on lesion size and attenuation, but also on acquisition parameters, patient habitus, image noise, and reconstruction method. These factors become particularly relevant when small calcified targets are evaluated or when dose reduction is desired, because image quality and radiation exposure must be balanced carefully [1–3].

Dual-energy computed tomography (CT) enables the generation of monoenergetic images with attenuation characteristics different from those of conventional polychromatic 120-kVp CT. Among these, 68-keV monoenergetic images are frequently used in clinical practice and are often compared with standard 120-kVp CT. However, the relationship between 68-keV monoenergetic imaging and conventional 120-kVp CT cannot be fully described by visual similarity alone. Quantitative image performance may differ depending on the metric considered, including attenuation, background noise, and contrast-to-noise ratio (CNR), and may further depend on phantom size and reconstruction method [4–8].

Advances in CT reconstruction, especially iterative reconstruction (IR) and deep learning-based reconstruction, further complicate this comparison. Compared with filtered back projection (FBP), these methods may substantially reduce image noise and alter lesion conspicuity. Consequently, the relative performance of 68-keV monoenergetic, routine-dose 120-kVp, and low-dose 120-kVp CT should be interpreted in the context of both reconstruction algorithm and imaging task [9–14].

For calcium-focused imaging, comparison based on CNR alone may be incomplete because radiation dose also differs across acquisition strategies. A higher CNR does not necessarily indicate greater dose efficiency if the corresponding volume CT dose index (CTDIvol) is also higher. Therefore, dose-normalized assessment using a figure of merit (FOM) may provide a more balanced comparison among 68-keV monoenergetic, routine-dose, and low-dose 120-kVp CT. In addition, phantom size is particularly relevant because increased attenuation and scatter may degrade image quality and alter the trade-off between conspicuity and dose [4–6, 15–18].

Therefore, the purpose of this study was to perform a quantitative comparison of 68-keV monoenergetic, routine-dose 120-kVp, and low-dose 120-kVp CT for calcium-mimicking inserts in thoracic phantoms. Specifically, we evaluated the effects of phantom size (without ring *versus* with ring) and reconstruction method (FBP, IR, and deep-learning reconstructionn (DL)) on attenuation, background noise, CNR, and dose-normalized performance expressed as FOM. The primary quantitative analysis focused on 5-mm and 3-mm inserts, whereas 1-mm inserts were evaluated exploratorily, given their expected susceptibility to partial volume effects at a reconstructed slice thickness of 2.5 mm. The present work should be interpreted primarily as a technical phantom-based comparison relevant to calcium-focused protocol optimization rather than as direct clinical validation.

## Materials and methods

### Study design

This phantom-based experimental study compared the quantitative performance of 68-keV monoenergetic, routine-dose 120-kVp, and low-dose 120-kVp CT in thoracic phantom configurations of different effective sizes. Ethics approval was not required because no human participants, human data, or animal data were involved.

### Phantom configuration and calcium-mimicking inserts

A QRM thorax phantom (QRM GmbH, Möhrendorf, Germany) was used to simulate thoracic anatomy. The phantom had external dimensions of approximately 200 × 300 mm and a height of 100 mm, with a central bore hole designed to accommodate inserts. A QRM cardiac calcification insert (CCI D100) was positioned in the central bore hole to simulate calcium-containing vascular structures. This configuration was defined as the small-phantom condition (without a ring).

To simulate a larger body habitus, a QRM extension ring (L size) was added around the thorax phantom. This configuration was defined as the large-phantom condition (with ring). The extension ring, composed of fat-equivalent material, increased the effective phantom cross-sectional area.

The CCI insert contained nine calcium-mimicking cylindrical inserts with three hydroxyapatite (HA) densities (800, 400, and 200 mg HA/cm³) and three nominal sizes (5, 3, and 1 mm in diameter, with corresponding lengths of 5, 3, and 1 mm, respectively). These inserts were used to simulate calcified vascular targets of different sizes and attenuation levels.

### CT acquisition and reconstruction

CT scans were performed using a GE Revolution Apex scanner (GE HealthCare, Waukesha, WI, USA). Three acquisition strategies were evaluated: routine-dose 120-kVp CT, low-dose 120-kVp CT, and dual-energy CT with 68-keV monoenergetic reconstruction.

Dual-energy scanning was performed using rapid kV switching between 80 and 140 kVp, followed by monoenergetic image reconstruction at 68 keV. The 68-keV monoenergetic level was selected because it represented the predefined monoenergetic reconstruction setting used in the present workflow and is frequently discussed in relation to standard 120-kVp CT in prior literature. Detailed acquisition parameters, noise index (NI) settings, and CTDIvol values for each protocol and phantom condition are summarized in Table 1.

**Table 1:** Scan parameters, noise index settings, and CTDIvol for routine-dose, low-dose, and dual-energy CT acquisitions in the small and large phantom configurations

Protocol^a^	Phantom^b^	Tube voltage/mono-energy	Smart mA	Noise index	Focal spot^c^	CTDIvol (mGy)^d^
Low-dose	Small	120 kVp	10–90	45.7	S	0.40
Low-dose	Large	120 kVp	70–500	55.0	S	1.51
Routine-dose	Small	120 kVp	20–650	43.1	S	2.39
Routine-dose	Large	120 kVp	35–900	49.8	S	4.05
Dual-energy	Small	68 keV^e^	145	38.2	L	4.57
Dual-energy	Large	68 keV	405	38.2	L	9.93

^a^ All protocols had a slice thickness of 2.5 mm, a pitch of 0.992:1, and a rotation time of 0.5 s

^b^ The phantom without a ring represents the small configuration, whereas the phantom with a ring represents the large configuration

^c^ S indicates a small focal spot, and L indicates a large focal spot

^d^ FBP, IR, and DL were reconstructed from the same raw data acquisition; therefore, CTDIvol was identical across reconstruction methods within each protocol and phantom condition

^e^ Dual-energy images were acquired using rapid kV switching between 80 and 140 kVp and subsequently reconstructed as 68-keV monoenergetic images

All images were reconstructed at a slice thickness of 2.5 mm using a standard kernel and three reconstruction methods: FBP, ASiR-V 50% IR, and TrueFidelity DLIR, medium strength (DL). All protocol groups were reconstructed at 2.5-mm slice thickness to maintain internal consistency across acquisition strategies and reconstruction methods within the present comparative framework. Because FBP, IR, and DL images were generated from the same raw data acquisition, CTDIvol was identical across reconstruction methods within each protocol and phantom condition [4, 5, 9, 13].

### Image selection

For each acquisition protocol, phantom condition, and reconstruction method, two slices were selected from each image series. These represented the slices showing the highest conspicuity of the calcium-mimicking inserts and were referenced by their original DICOM filenames to ensure reproducibility and avoid ambiguity related to slice ordering. Slice selection and region of interest (ROI) center determination were performed by the same investigator and then applied consistently across the corresponding reconstructions from the same acquisition.

### ROI analysis

ROI placement was performed using ImageJ (National Institutes of Health, Bethesda, MD, USA). For each selected slice, nine circular ROIs were placed at the centers of the calcium-mimicking inserts, corresponding to the three HA densities and three insert sizes. In addition, one large background ROI was placed in a visually homogeneous region of the insert background, with exclusion circles applied to avoid contamination by the calcification inserts and their immediate surroundings.

ROI definitions were held constant within each scan condition and applied consistently across both selected slices. A custom Python-based workflow was then used to read the DICOM images directly, convert pixel values to Hounsfield units (HU) using the DICOM rescale slope and intercept, and extract ROI statistics [10, 11].

### Quantitative measurements

For each insert ROI and background ROI, the following parameters were extracted: ROI area in mm² (area_mm²), mean attenuation in Hounsfield units (mean HU), and standard deviation (SD). Background noise was defined as the standard deviation of the background ROI. CNR was defined as the absolute difference between the mean attenuation of the insert ROI and that of the background ROI, divided by the standard deviation of the background ROI. To account for radiation dose, a FOM was calculated as:

$${{{\rm{FOM}}}}={{{\rm{CN}}}}{{{{\rm{R}}}}}^{2}/{{{\rm{CTDIvol}}}}.$$

Thus, CNR was used to characterize lesion conspicuity, whereas FOM was used to evaluate dose-normalized quantitative performance.

### Primary and exploratory analysis targets

The primary quantitative analysis focused on the 5-mm and 3-mm calcium-mimicking inserts because these targets were more consistently visualized and quantified on images reconstructed at 2.5-mm slice thickness. The 1-mm inserts were analyzed only as an exploratory target, because their extremely small size relative to the reconstructed slice thickness made them highly susceptible to partial volume effects and unstable attenuation measurements.

### Comparative framework

Quantitative comparisons were performed across the three acquisition strategies: 68-keV monoenergetic CT, routine-dose 120-kVp CT, and low-dose 120-kVp CT. For each insert size and HA density, comparisons were stratified by phantom condition (without ring *versus* with ring) and reconstruction method (FBP, IR, DL).

The primary interpretation focused on the relationship among attenuation, background noise, CNR, CTDIvol, and FOM. In particular, the analysis was designed to determine whether higher lesion conspicuity was accompanied by higher radiation dose, and whether the observed differences among acquisition strategies should be interpreted in the context of protocol-dependent noise targets and dose settings.

### Statistical analysis

Quantitative measurements were summarized descriptively for each acquisition protocol, phantom condition, reconstruction method, insert size, and HA density. For the main manuscript tables, values from the two selected slices were averaged for each condition. Repeated scans and repeated ROI redraws were not performed in the present study. Because this was a phantom-based technical study and the primary objective was to compare attenuation, noise, CNR, and FOM across imaging conditions, the primary analysis was descriptive [10, 19]. Accordingly, statements describing relative performance among acquisition strategies should be interpreted as descriptive comparisons rather than as formally tested statistical differences. The 5-mm and 3-mm inserts were treated as the primary analysis targets, whereas the 1-mm inserts were treated as exploratory due to their limited measurement stability.

### Use of a large language model for assistance

Large language model assistance was used only for language refinement and manuscript organization. It was not used for study design, data generation, data analysis, statistical analysis, or scientific interpretation. All scientific content, analysis, interpretation, and final manuscript decisions remained the responsibility of the authors.

## Results

### Overall quantitative trends

Quantitative measurements were successfully obtained for all three acquisition protocols, both phantom configurations, and all three reconstruction methods. For the 5-mm and 3-mm calcium-mimicking inserts, measured attenuation generally increased with increasing hydroxyapatite (HA) density, supporting the consistency of ROI placement and analysis. In contrast, the 1-mm inserts showed substantially less stable behavior and were therefore treated as exploratory rather than primary quantitative targets.

Across nearly all conditions, the with-ring configuration showed higher background noise and lower CNR than the corresponding without-ring configuration. Reconstruction-dependent differences were also consistently observed, with DL generally providing the lowest background noise and the highest CNR, followed by IR, whereas FBP showed the highest noise and the lowest CNR [10].

### Quantitative results for 5-mm inserts

**Fig. 1:**
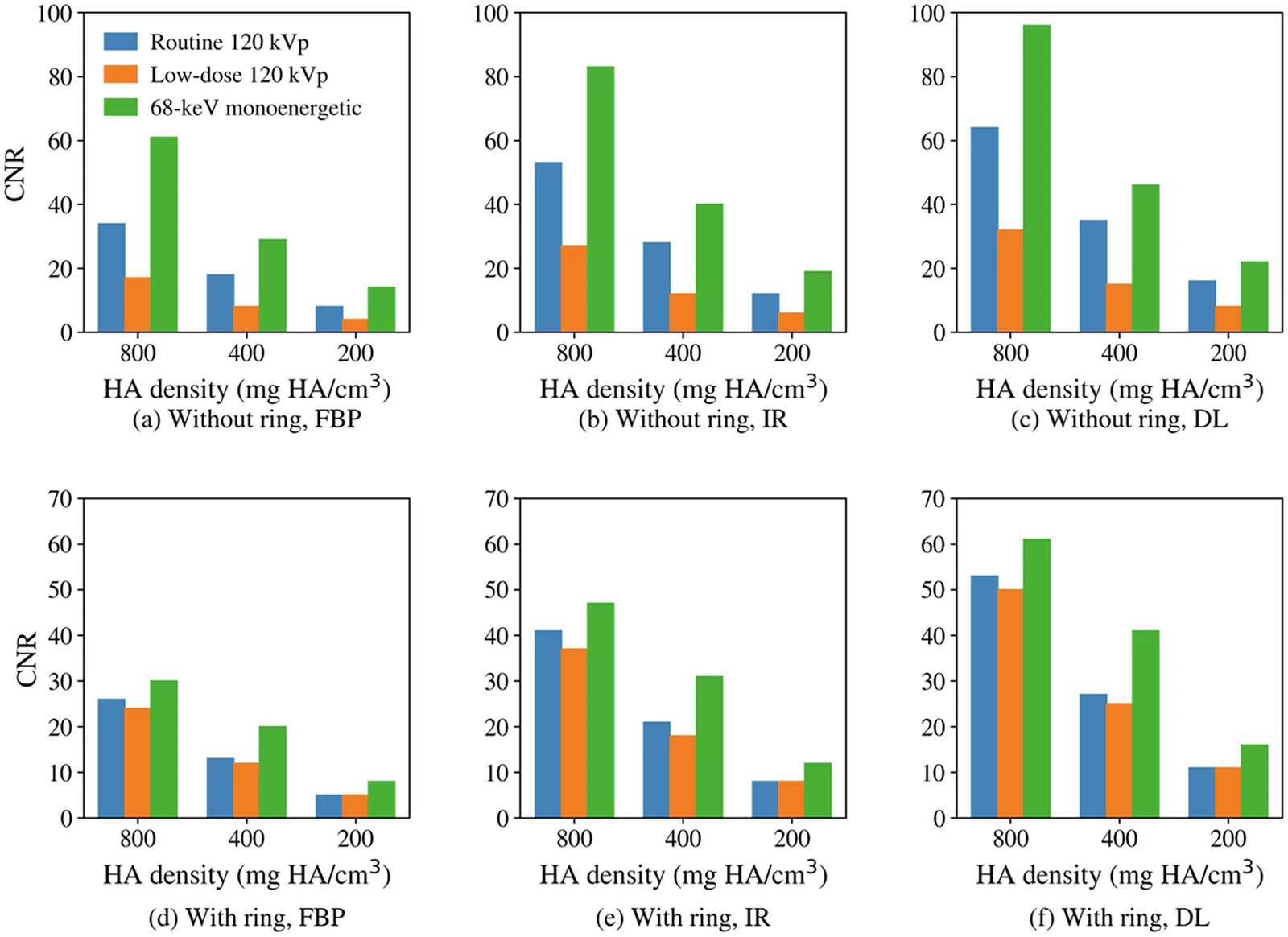
Quantitative comparison of contrast-to-noise ratio (CNR) for 5-mm calcium-mimicking inserts. CNR values are shown for three hydroxyapatite densities (800, 400, and 200 mg HA/cm³) under routine-dose 120-kVp CT, low-dose 120-kVp CT, and dual-energy CT with 68-keV monoenergetic reconstruction. **a**–**c** correspond to the without-ring condition reconstructed with FBP, IR, and DL, respectively, whereas (**d**–**f**) correspond to the with-ring condition. For each acquisition condition, the plotted values represent the average of the two selected slices

The quantitative results for the 5-mm inserts are summarized in Table 2, and the corresponding CNR trends are illustrated in Fig. 1. For all three acquisition strategies, attenuation and CNR were highest for the 800 mg HA/cm³ inserts, intermediate for the 400 mg HA/cm³ inserts, and lowest for the 200 mg HA/cm³ inserts, indicating a density-dependent response across reconstruction methods.

**Table 2:** Quantitative results for 5-mm calcium-mimicking inserts. Values represent the average of the two selected slices for each condition

Protocol	Phantoms	Recon^a^	Density mg HA/cm³	Mean HU	Background noise^b^	CNR	FOM^c^
Low-dose	Small	DL	800	682	20	32	2,812
Low-dose	Small	DL	400	337	20	15	655
Low-dose	Small	DL	200	200	20	8	169
Low-dose	Small	FBP	800	679	37	17	812
Low-dose	Small	FBP	400	327	37	8	180
Low-dose	Small	FBP	200	199	37	4	48
Low-dose	Small	IR	800	675	24	27	1,927
Low-dose	Small	IR	400	321	24	12	420
Low-dose	Small	IR	200	190	24	6	102
Low-dose	Large	DL	800	657	12	50	1,057
Low-dose	Large	DL	400	349	12	25	261
Low-dose	Large	DL	200	178	12	11	51
Low-dose	Large	FBP	800	668	26	24	248
Low-dose	Large	FBP	400	347	26	12	59
Low-dose	Large	FBP	200	184	26	5	13
Low-dose	Large	IR	800	666	17	37	558
Low-dose	Large	IR	400	346	17	18	132
Low-dose	Large	IR	200	175	17	8	26
Routine-dose	Small	DL	800	683	10	64	2,931
Routine-dose	Small	DL	400	395	10	35	830
Routine-dose	Small	DL	200	204	10	16	185
Routine-dose	Small	FBP	800	687	19	34	830
Routine-dose	Small	FBP	400	390	19	18	225
Routine-dose	Small	FBP	200	195	19	8	47
Routine-dose	Small	IR	800	685	12	53	2,013
Routine-dose	Small	IR	400	387	12	28	541
Routine-dose	Small	IR	200	191	12	12	107
Routine-dose	Large	DL	800	789	14	53	610
Routine-dose	Large	DL	400	422	14	27	158
Routine-dose	Large	DL	200	201	14	11	27
Routine-dose	Large	FBP	800	786	29	26	144
Routine-dose	Large	FBP	400	421	29	13	37
Routine-dose	Large	FBP	200	201	29	5	6
Routine-dose	Large	IR	800	786	18	41	367
Routine-dose	Large	IR	400	421	18	21	95
Routine-dose	Large	IR	200	196	18	8	15
Dual-energy	Small	DL	800	893	9	96	2,269
Dual-energy	Small	DL	400	451	9	46	547
Dual-energy	Small	DL	200	231	9	22	116
Dual-energy	Small	FBP	800	889	14	61	932
Dual-energy	Small	FBP	400	443	14	29	218
Dual-energy	Small	FBP	200	230	14	14	48
Dual-energy	Small	IR	800	890	10	83	1,721
Dual-energy	Small	IR	400	442	10	40	399
Dual-energy	Small	IR	200	228	10	19	86
Dual-energy	Large	DL	800	653	10	61	434
Dual-energy	Large	DL	400	453	10	41	171
Dual-energy	Large	DL	200	196	10	16	26
Dual-energy	Large	FBP	800	644	20	30	103
Dual-energy	Large	FBP	400	442	20	20	39
Dual-energy	Large	FBP	200	200	20	8	7
Dual-energy	Large	IR	800	639	13	47	257
Dual-energy	Large	IR	400	438	13	31	97
Dual-energy	Large	IR	200	194	13	12	15

^a^ FBP, IR, and DL denote filtered back projection, ASiR-V 50% iterative reconstruction, and TrueFidelity DLIR (medium strength), respectively

^b^ Background noise was defined as the standard deviation of the background ROI

^c^ FOM was calculated as CNR²/CTDIvol

In the without-ring condition, 68-keV imaging generally yielded the highest CNR, followed by routine-dose and low-dose 120-kVp CT. Among the three reconstruction methods, DL consistently produced the highest CNR and FOM, whereas FBP yielded the lowest FOM because of its higher background noise. For example, in the without-ring condition at 800 mg HA/cm³, CNR increased from approximately 34.0 with routine-dose FBP to 61.3 with 68-keV FBP, and from 63.9 with routine-dose DL to 95.7 with 68-keV DL. As shown in Fig. 1, this ranking was preserved across the three acquisition strategies.

In the with-ring condition, quantitative performance decreased for all protocols and reconstructions. Nevertheless, the same general pattern remained, with DL > IR > FBP, and with 68-keV imaging generally producing the highest CNR. However, this CNR advantage must be interpreted together with radiation dose, because CTDIvol was highest for the 68-keV acquisition, intermediate for routine-dose 120-kVp CT, and lowest for low-dose 120-kVp CT. Accordingly, although 68-keV imaging yielded the highest CNR, the corresponding differences in FOM were smaller than the CNR differences alone would suggest [15, 18].

### Quantitative results for 3-mm inserts

**Fig. 2:**
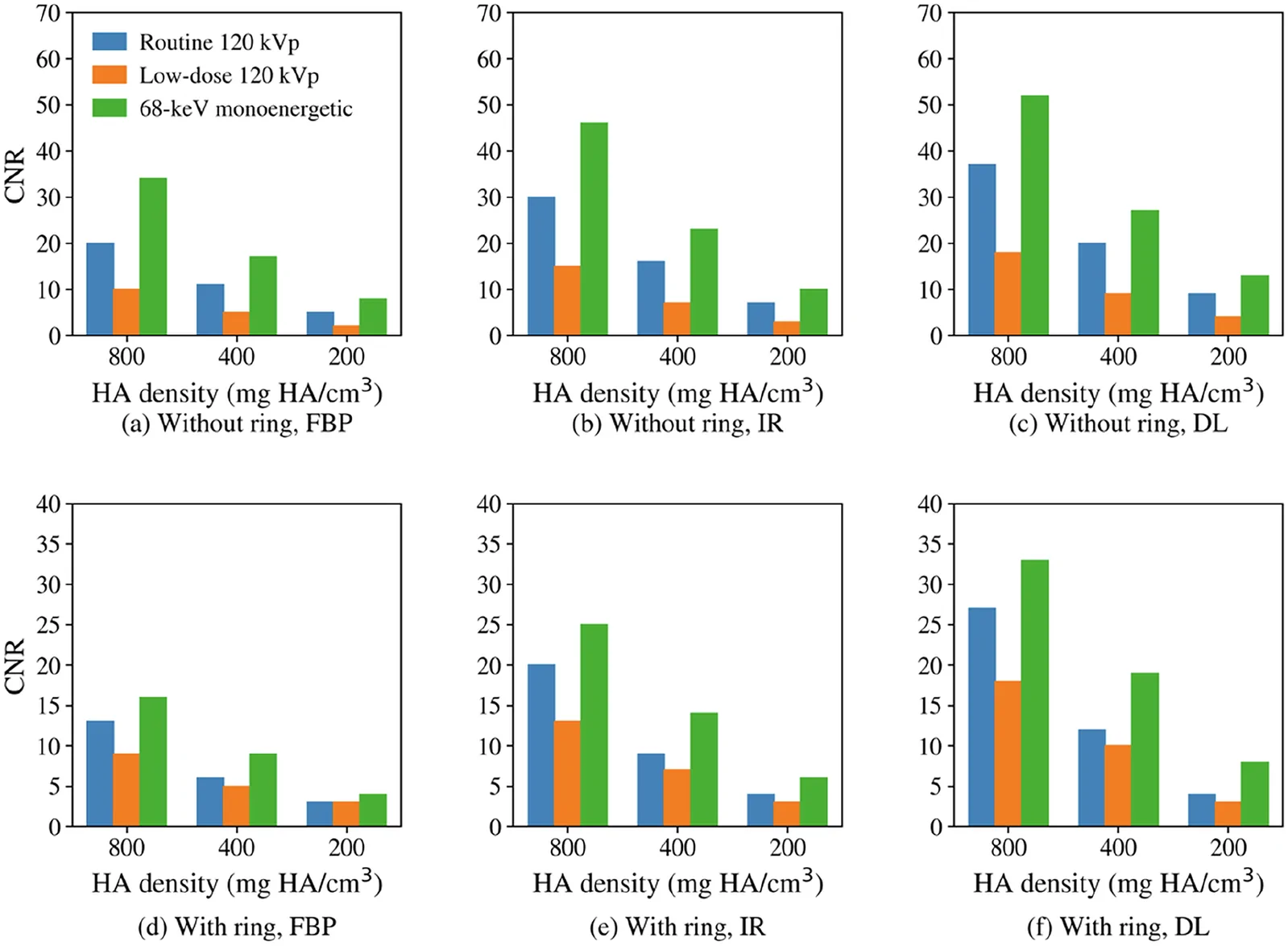
Quantitative comparison of contrast-to-noise ratio (CNR) for 3-mm calcium-mimicking inserts. CNR values are shown for three hydroxyapatite densities (800, 400, and 200 mg HA/cm³) under routine-dose 120-kVp CT, low-dose 120-kVp CT, and dual-energy CT with 68-keV monoenergetic reconstruction. **a**–**c** correspond to the without-ring condition reconstructed with FBP, IR, and DL, respectively, whereas (**d**–**f**) correspond to the with-ring condition. For each acquisition condition, the plotted values represent the average of the two selected slices

The quantitative results for the 3-mm inserts are summarized in Table 3, and the corresponding CNR trends are illustrated in Fig. 2. As expected, measured attenuation and CNR were lower than those observed for the 5-mm inserts under otherwise matched conditions, reflecting the smaller target size and greater susceptibility to partial-volume effects.

**Table 3:** Quantitative results for 3-mm calcium-mimicking inserts. Values represent the average of the two selected slices for each condition

Protocol	Phantoms	Recon^a^	Density mg HA/cm³	Mean HU	Background noise^b^	CNR	FOM^c^
Low-dose	Small	DL	800	398	20	18	1,375
Low-dose	Small	DL	400	214	20	9	361
Low-dose	Small	DL	200	119	20	4	63
Low-dose	Small	FBP	800	396	37	10	397
Low-dose	Small	FBP	400	211	37	5	108
Low-dose	Small	FBP	200	122	37	2	21
Low-dose	Small	IR	800	388	24	15	939
Low-dose	Small	IR	400	204	24	7	236
Low-dose	Small	IR	200	114	24	3	40
Low-dose	Large	DL	800	262	12	18	134
Low-dose	Large	DL	400	162	12	10	39
Low-dose	Large	DL	200	78	12	3	3
Low-dose	Large	FBP	800	273	26	9	34
Low-dose	Large	FBP	400	172	26	5	11
Low-dose	Large	FBP	200	109	26	3	3
Low-dose	Large	IR	800	267	17	13	73
Low-dose	Large	IR	400	163	17	7	21
Low-dose	Large	IR	200	100	17	3	5
Routine-dose	Small	DL	800	410	10	37	1,081
Routine-dose	Small	DL	400	244	10	20	280
Routine-dose	Small	DL	200	135	10	9	67
Routine-dose	Small	FBP	800	416	19	20	311
Routine-dose	Small	FBP	400	248	19	11	82
Routine-dose	Small	FBP	200	135	19	5	18
Routine-dose	Small	IR	800	411	12	30	744
Routine-dose	Small	IR	400	239	12	16	182
Routine-dose	Small	IR	200	128	12	7	39
Routine-dose	Large	DL	800	423	14	27	172
Routine-dose	Large	DL	400	210	14	12	34
Routine-dose	Large	DL	200	104	14	4	4
Routine-dose	Large	FBP	800	423	29	13	41
Routine-dose	Large	FBP	400	213	29	6	9
Routine-dose	Large	FBP	200	134	29	3	2
Routine-dose	Large	IR	800	405	18	20	98
Routine-dose	Large	IR	400	202	18	9	20
Routine-dose	Large	IR	200	121	18	4	4
Dual-energy	Small	DL	800	503	9	52	672
Dual-energy	Small	DL	400	275	9	27	185
Dual-energy	Small	DL	200	149	9	13	39
Dual-energy	Small	FBP	800	506	14	34	282
Dual-energy	Small	FBP	400	271	14	17	74
Dual-energy	Small	FBP	200	148	14	8	16
Dual-energy	Small	IR	800	504	10	46	514
Dual-energy	Small	IR	400	268	10	23	132
Dual-energy	Small	IR	200	145	10	10	27
Dual-energy	Large	DL	800	366	10	33	197
Dual-energy	Large	DL	400	227	10	19	45
Dual-energy	Large	DL	200	118	10	8	11
Dual-energy	Large	FBP	800	370	20	16	48
Dual-energy	Large	FBP	400	229	20	9	11
Dual-energy	Large	FBP	200	124	20	4	3
Dual-energy	Large	IR	800	360	13	25	117
Dual-energy	Large	IR	400	219	13	14	26
Dual-energy	Large	IR	200	112	13	6	6

^a^ FBP, IR, and DL denote filtered back projection, ASiR-V 50% iterative reconstruction, and TrueFidelity DLIR (medium strength), respectively

^b^ Background noise was defined as the standard deviation of the background ROI

^c^ FOM was calculated as CNR²/CTDIvol

Despite this reduction, the 3-mm inserts still showed consistent density-dependent trends across most conditions. In both phantom configurations, DL again produced the most favorable quantitative performance, with higher CNR and FOM than IR and FBP. For example, in the without-ring condition at 800 mg HA/cm³, CNR increased from approximately 19.7 with routine-dose FBP to 33.8 with 68-keV FBP, and from 36.7 with routine-dose DL to 52.1 with 68-keV DL. In the with-ring condition, the corresponding CNR values were lower across all three acquisition strategies, illustrating the adverse effect of increased phantom size.

Similar to the 5-mm results, 68-keV imaging generally yielded the highest CNR for 3-mm inserts, whereas low-dose 120-kVp CT yielded the lowest. However, once CTDIvol was taken into account, the differences in FOM among 68-keV, routine-dose, and low-dose acquisitions became less pronounced. Thus, for the 3-mm inserts, the relative advantage of 68-keV imaging was more evident for CNR than for dose-normalized performance [15, 18].

### Comparison of CNR, dose, and FOM among acquisition strategies

When the three acquisition strategies were compared directly, several consistent patterns emerged. First, the without-ring configuration provided lower background noise and higher CNR than the with-ring configuration across all protocols, demonstrating the expected adverse effect of increased phantom size. Second, DL reconstruction consistently improved CNR and FOM relative to both IR and FBP, regardless of acquisition protocol. Third, the ranking of CNR was generally: 68-keV > routine-dose 120-kVp > low-dose 120-kVp CT.

However, CTDIvol followed the same general order: 68-keV > routine-dose 120-kVp > low-dose 120-kVp CT.

As a result, the observed CNR advantage of 68-keV imaging was attenuated when performance was normalized to dose using FOM. These trends are visually summarized in Figs. 1 and 2 and numerically summarized in Tables 2 and 3. Taken together, the data indicate that 68-keV monoenergetic imaging generally provides the highest lesion conspicuity, but that the overall dose-normalized performance of the three acquisition strategies is more similar than CNR alone would imply.

### Exploratory results for 1-mm inserts

The 1-mm inserts showed considerably less stable quantitative behavior than the 5-mm and 3-mm inserts. In several conditions, measured attenuation values did not follow the expected monotonic ranking with increasing HA density, particularly in the larger phantom configuration. This instability is likely attributable to severe through-plane partial-volume averaging at the 2.5-mm slice thickness, together with the extremely small physical size of the target [19].

Because of this limited stability, the 1-mm results were not considered sufficiently robust for primary quantitative comparisons. Instead, they were treated as exploratory observations supporting the interpretation that 2.5-mm reconstructed images are not optimal for reliable quantitative assessment of calcification-mimicking targets of this size.

### Summary of main findings

Overall, the quantitative analysis showed that 68-keV monoenergetic imaging generally provided the highest CNR for 5-mm and 3-mm inserts, whereas low-dose 120-kVp CT generally provided the lowest. Quantitative performance was consistently influenced by phantom size and reconstruction method, with the with-ring condition reducing CNR and FOM and DL reconstruction providing the most favorable results. However, because CTDIvol was also highest for the 68-keV acquisition, FOM values among 68-keV, routine-dose 120-kVp, and low-dose 120-kVp CT were more comparable than the CNR results alone would suggest.

## Discussion

The present study compared dual-energy CT-derived 68-keV monoenergetic images with routine-dose and low-dose 120-kVp CT for calcium-mimicking inserts in thoracic phantom configurations of different effective sizes. The main findings can be summarized as follows. First, for the 5-mm and 3-mm inserts, 68-keV imaging generally produced the highest CNR, followed by routine-dose and low-dose 120-kVp CT. Second, the observed CNR advantage of 68-keV imaging was accompanied by higher radiation dose, such that FOM values across the three acquisition strategies were more similar than the CNR values alone would suggest. Third, phantom size strongly affected quantitative performance, with the with-ring condition consistently increasing background noise and reducing CNR and FOM. Fourth, the reconstruction method had a marked effect on quantitative performance, with DL generally providing the most favorable results. Finally, the 1-mm inserts showed limited quantitative stability at 2.5-mm slice thickness and were not suitable as primary analysis targets [4–6].

One motivation for this study was the common assumption that 68-keV monoenergetic images approximate conventional 120-kVp CT. The present results suggest that this formulation is incomplete. In our data, 68-keV imaging was not merely similar to routine-dose 120-kVp CT; rather, it generally produced higher CNR for both 5-mm and 3-mm inserts. Accordingly, the relationship between 68-keV and conventional 120-kVp CT is better understood as a trade-off between lesion conspicuity and radiation dose rather than as simple visual equivalence. The present work should therefore be interpreted as a technical phantom-based comparison relevant to calcium-focused protocol optimization rather than as direct clinical validation. This interpretation is consistent with the concept that monoenergetic image behavior is influenced not only by effective energy, but also by acquisition noise and reconstruction characteristics [4, 6, 15].

The comparison with low-dose 120-kVp CT further supports this interpretation. Low-dose CT consistently produced lower CTDIvol, but also lower CNR. Routine-dose 120-kVp CT occupied an intermediate position between low-dose 120-kVp CT and 68-keV monoenergetic imaging for both dose and CNR. This pattern indicates that the three acquisition strategies form a dose-performance continuum rather than a binary comparison. In this framework, 68-keV imaging offers improved conspicuity, but at a higher dose cost, whereas low-dose 120-kVp CT reduces dose at the expense of lower lesion conspicuity [15, 17, 18].

The observed differences in CNR among 68-keV monoenergetic, routine-dose, and low-dose 120-kVp CT should also be interpreted in the context of protocol design. In the present study, the dual-energy protocol had the lowest noise index (NI) and the highest CTDIvol, whereas the low-dose protocol had the highest NI and the lowest CTDIvol. Accordingly, the descriptively higher CNR observed with 68-keV imaging cannot be attributed solely to energy-dependent image characteristics, but should also be understood in relation to protocol-dependent noise targets and dose settings. This suggests that the relative ranking of noise and CNR across acquisition strategies may be protocol-dependent rather than fixed.

A key implication of the present data is that CNR alone does not fully capture the relative performance of the three acquisition strategies. Because CTDIvol was highest for the 68-keV acquisition, the observed CNR advantage of 68-keV imaging in Figs. 1 and 2 should be interpreted alongside the FOM results in Tables 2 and 3. Once the dose was taken into account, the differences among 68-keV, routine-dose, and low-dose CT became smaller. Thus, the present findings do not indicate an overall advantage of 68-keV imaging across all performance metrics; rather, they suggest that 68-keV imaging provides higher CNR, whereas the dose-normalized efficiency of the three strategies is more comparable.

The reconstruction-dependent findings were also consistent across protocols. DL generally yielded lower background noise and higher CNR than IR and FBP, and it also produced the most favorable FOM values under most conditions. This observation is consistent with previous reports indicating that advanced reconstruction methods can improve image quality by reducing noise while preserving relevant signal characteristics [9, 11, 13]. In practical terms, this means that conclusions regarding the relative performance of 68-keV and 120-kVp CT should always be interpreted in the context of the reconstruction method.

Phantom size had a clear and systematic effect. The with-ring configuration was associated with degraded quantitative performance, as reflected by increased background noise and reduced CNR and FOM. This finding is physically plausible, given the increased attenuation and scatter introduced by the extension ring. As illustrated in Figs. 1 and 2, the penalty associated with increased phantom size was evident across all three reconstruction methods, although DL preserved CNR more effectively than IR and FBP. These results indicate that the balance between conspicuity and dose becomes less favorable as effective phantom size increases [4, 5, 10].

The 1-mm inserts require separate consideration. Their behavior was substantially less stable than that of the 5-mm and 3-mm inserts, and attenuation ranking across HA densities was not consistently preserved. This instability is most likely due to severe partial volume effects, as the nominal 1-mm insert length is markedly smaller than the 2.5-mm reconstructed slice thickness. These findings indicate that reliable quantitative assessment of targets of this size would likely require thinner reconstructions and/or a dedicated high-resolution protocol [10, 19, 20].

This study has several limitations. First, it was a phantom-based investigation conducted on a single CT platform using a single set of reconstruction parameters; therefore, the results may be vendor- and system-specific. Second, the primary analysis was descriptive and focused on quantitative image metrics rather than observer-based detection performance, and neither repeated scans nor repeated ROI redraws were performed. Third, only one monoenergetic level, 68 keV, was evaluated in detail, although other keV levels may also be relevant for optimization. Fourth, the acquisition strategies were not dose-matched, making it difficult to isolate the effect of monoenergetic imaging itself from the influence of radiation dose and noise target settings. Fifth, the exclusive use of 2.5-mm reconstructions likely increased partial-volume effects for the smaller inserts and limits direct extrapolation to applications in which thinner reconstructions are routinely used. Finally, the 1-mm inserts were not suitable for robust primary analysis at the present reconstruction thickness.

Despite these limitations, the study provides a structured comparison among routine-dose 120-kVp CT, low-dose 120-kVp CT, and dual-energy CT with 68-keV monoenergetic reconstruction under controlled phantom conditions. The inclusion of two phantom sizes and three reconstruction methods, together with dose-normalized assessment using FOM, strengthens the technical relevance of the findings and provides a useful framework for understanding how monoenergetic imaging behaves relative to conventional acquisitions in calcium-focused CT tasks. Rather than asking only whether 68-keV imaging is similar to routine-dose 120-kVp CT, the present data suggest that the more relevant question is how lesion conspicuity, radiation dose, and dose-normalized performance should be balanced across acquisition strategies. Viewed in this way, 68-keV monoenergetic imaging can provide higher CNR, whereas routine-dose and low-dose 120-kVp CT remain competitive when performance is evaluated in terms of dose.

For 5-mm and 3-mm calcium-mimicking inserts, 68-keV monoenergetic imaging generally provided the highest CNR, followed by routine-dose and low-dose 120-kVp CT. However, this advantage was accompanied by higher CTDIvol. When dose was accounted for using FOM, the quantitative performance of the three acquisition strategies became more comparable. Phantom size and reconstruction method substantially influenced this trade-off, with the with-ring condition reducing performance and DL yielding the most favorable overall results. In contrast, 1-mm insert measurements were unstable at 2.5-mm slice thickness and should be interpreted as exploratory. Because this was a phantom-based technical study, clinical validation remains necessary before these findings can be translated into routine calcium-focused CT tasks or patient-specific protocol selection. Further studies across additional monoenergetic energy levels, CT vendors, reconstruction platforms, and patient-based datasets are warranted to confirm the generalizability and clinical relevance of these descriptive findings.

## Data Availability

The datasets used and/or analyzed during the current study are available from the corresponding author on reasonable request.

## Abbreviations

CNR: Contrast-to-noise ratio
CT: Computed tomography
CTDIvol: Volume CT dose index
DL: Deep-learning reconstruction
FBP: Filtered back projection
FOM: Figure of merit
HA: Hydroxyapatite
IR: Iterative reconstruction
NI: Noise index
ROI: Region of interest
